# Assessment of Undiagnosed Cardiovascular Risk Factors Identified Through the Saudi Model of Care Screening Program in the Qassim Region, Saudi Arabia

**DOI:** 10.7759/cureus.105362

**Published:** 2026-03-17

**Authors:** Fatmah Alribdi, Mohammed Awad Alanazi, Abdarrahman Albhairy, Abdulrahman Mesned, Annalyn V Camba, Ahmed A Almeman, Abdulsalam A Alfawzan, Khuzama Alkhalaf, Albandary Alanazi, Abdullah Al Qwaee

**Affiliations:** 1 Saudi Model of Care, Qassim Health Cluster, Buraidah, SAU; 2 Pediatric Cardiology, Prince Sultan Cardiac Centre, Buraidah, SAU; 3 Pharmacology, College of Medicine, Qassim University, Buraidah, SAU

**Keywords:** cardiovascular disease, diabetes mellitus, epidemiology, hypertension, obesity, prevalence, risk factors

## Abstract

Background: Saudi Arabia is experiencing an increasing prevalence of undiagnosed chronic conditions, like diabetes, hypertension, obesity, and dyslipidemia. In line with national health initiatives, the Saudi Model of Care (SMoC) launched an early screening program in Qassim to identify undiagnosed cases and inform healthcare planning. This study aimed to evaluate the effectiveness of the SMoC screening program in detecting cardiometabolic disorders among individuals without prior diagnoses in Qassim, Saudi Arabia.

Methods: A cross-sectional study design was employed. Analyses used data from 7,359 adults screened at primary healthcare centers in Qassim. Data collection encompassed anthropometric measures, glycated hemoglobin (HbA1c), blood pressure, and lipid profiles, all categorized using established standard thresholds. Statistical analyses included descriptive analyses and Chi-square tests.

Results: The screening initiative evaluated 7,359 participants (mean age 38.0 ± 12.4 years; 59.8% female). The cohort exhibited a generally normotensive profile; however, a significant burden of previously undiagnosed metabolic risk factors was identified. Key findings indicated that 4,389 (59.6%) participants presented with dyslipidemia, 708 (9.6%) had diabetes (HbA1c > 6.5%), 2,039 (27.7%) were classified as obese (BMI > 30 kg/m^2^), and 3,830 (52.0%) reported insufficient physical activity. Furthermore, Chi-square analyses indicated no statistically significant associations between physical activity levels and either diabetes diagnosis or obesity status (p > 0.05).

Conclusion: Population-level screening in primary care settings uncovers a critical volume of undiagnosed cardiometabolic diseases, predominantly dyslipidemia and diabetes. Shifting from symptom-triggered testing to proactive, routine screening is essential for early intervention and mitigating long-term cardiovascular morbidity in this demographic.

## Introduction

Chronic non-communicable diseases (NCDs) constitute a significant global health crisis, responsible for 74% of worldwide deaths and imposing severe social and economic burdens across nations [1]. The World Health Organization (WHO) identifies cardiovascular diseases, diabetes, cancer, and chronic respiratory diseases as the principal contributors to this burden [2]. These conditions devastate individual lives through prolonged disability and premature death while also straining economies through elevated healthcare costs and a loss of productivity. The epidemiological transition toward NCD dominance has been particularly accelerated in Gulf Cooperation Council countries, where rapid urbanization, sedentary lifestyles, and dietary shifts have converged to create a perfect storm of chronic disease risk.

Saudi Arabia exemplifies this troubling transition, where NCDs, particularly diabetes, dyslipidemia (defined as ≥1 abnormal lipid parameter), hypertension, and obesity, drive approximately 73.2% of all deaths nationally, ranking among the highest burdens in the Arabian Gulf region [3]. Cardiovascular diseases alone account for 37% of Saudi mortality, followed by cancer (10%) and diabetes (3%), creating a public health imperative that demands innovative, population-level solutions [2]. Alarmingly, an approximate 57.8% of cardiometabolic conditions remain undiagnosed until complications arise [4], leading to preventable morbidity in economically active populations.

The Saudi Arabian context reveals alarming epidemiological trends driven by sedentary lifestyles and socioeconomic factors. The kingdom has witnessed increases in metabolic disorders over recent decades, with diabetes prevalence surging to over 21% [5], alongside parallel rises in hypertension, affecting 26.1% of the adult population [3], and dyslipidemia, affecting 25.5% [6]. These trends are anchored in a complex interplay of behavioral and structural determinants where only 19% of Saudi adults meet WHO physical activity recommendations (defined as at least 150 min/week moderate activity), and dietary patterns have shifted toward excessive consumption of refined carbohydrates, saturated fats, and sugar-sweetened beverages.

In response to this mounting crisis, Saudi Vision 2030 has established healthcare transformation as a fundamental pillar of national development. Launched in 2016, this strategic framework recognizes that population health is fundamental to economic vitality and social progress [7]. The Healthcare Sector Transformation Program specifically addresses NCDs through ambitious initiatives: implementing universal health coverage, expanding infrastructure, and deploying innovative screening models like the Saudi Model of Care (SMoC) that prioritize early detection and proactive prevention to transform healthcare delivery. To operationalize the healthcare transformation outlined in Vision 2030, the Ministry of Health launched the SMoC, a patient-centered framework that reorients services from reactive treatment to proactive, preventive care. The SMoC is structured around six Systems of Care: Keeping Well, Planned Procedures, Women & Children, Urgent Problems, Chronic Conditions, and the Last Phase of Life, designed to deliver integrated, equitable services across primary, secondary, and tertiary levels. It emphasizes population health management, early risk identification, and continuity of care to reduce the burden of NCDs and improve long-term outcomes [8,9]. Moreover, previous research conducted under the SMoC framework in the Qassim region has reported measurable improvements in quality of life and patient experience among oncology palliative care recipients, illustrating the value of structured care pathways in enhancing health service outcomes [10]. Crucially, the strategy acknowledges that effective NCD control requires multisectoral coordination beyond traditional healthcare boundaries. The SMoC operationalizes this through integrated screening in primary care, targeting early detection of modifiable risks [11]. While international programs (e.g., UK NHS Health Check) demonstrate that screening can reduce long-term complications [12], regional data on undiagnosed NCDs in Saudi Arabia are sparse. This gap impedes resource allocation for prevention, particularly for dyslipidemia, subclinical diabetes, and obesity-driven metabolic dysfunction.

The Qassim screening initiative under the SMoC represents a pioneering application of Vision 2030's preventive health principles. By targeting apparently healthy adults without prior NCD diagnoses, this program addresses the critical challenge of undetected chronic conditions that silently progress toward complications. The region's epidemiological profile makes it a strategic priority: Qassim reports diabetes prevalence exceeding 27% among adults, significantly higher than the national average of 21%, and hypertension affecting over 23% of adults [5], yet community screening remains limited.

This study characterized undiagnosed conditions detected by the SMoC screening program in the Qassim region, focusing on dyslipidemia, dysglycemia, obesity, hypertension, and physical inactivity. By examining these epidemiological patterns and their lifestyle correlates, this research aims to provide empirical data to refine Saudi Arabia's NCD control strategies within the Vision 2030 framework, addressing region-specific health profiles and identifying hidden disease burdens.

## Materials and methods

Study design and sampling

We conducted a population-based cross-sectional analysis utilizing data from the SMoC screening program, collected between January 2025 and June 2025. The study was conducted in primary healthcare centers (PHCs) across Qassim's urban and rural districts. To ensure equitable geographical representation, these PHCs were selected utilizing a proportionate stratified random sampling approach based on the population size of each district. PHCs serve as frontline healthcare access points under Saudi Arabia's decentralized healthcare system, making them ideal for community-level screening.

Inclusion and exclusion criteria

Inclusion criteria comprised adults aged 18 years and older residing in the Qassim region who attended PHCs for routine services (e.g., vaccinations, wellness checks) during the study period. Eligible participants had no prior documented diagnosis of diabetes, hypertension, dyslipidemia, or obesity in the Health Information System (HIS). Conversely, exclusion criteria included pregnant women, non-residents of the Qassim region, and patients with a known prior diagnosis of the aforementioned chronic conditions. Additionally, individuals with terminal illnesses, those undergoing active cancer treatment, and patients with incomplete demographic or screening data were excluded from the analysis.

Clinical definitions and measurement tools

Clinical parameters were categorized based on established medical thresholds. Dyslipidemia was defined as possessing at least one abnormal lipid parameter: total cholesterol ≥ 5.2 mmol/L, low-density lipoprotein (LDL) cholesterol ≥ 2.6 mmol/L, high-density lipoprotein (HDL) cholesterol < 1.55 mmol/L, or triglycerides ≥ 1.7 mmol/L. Diabetes mellitus was diagnosed with a glycated hemoglobin (HbA1c) level > 6.5%, while severe (uncontrolled) diabetes was classified as HbA1c > 8.0%. Obesity was defined as a body mass index (BMI) > 30 kg/m^2^, and normal blood pressure (BP) was evaluated against the standard threshold of < 120/80 mmHg, with severe hypertension defined as > 150/100 mmHg. Furthermore, physical activity was assessed via a standardized self-reported questionnaire based on WHO guidelines, categorizing "sufficient" activity as engaging in at least 150 minutes of moderate-intensity exercise per week.

Data analysis

Continuous variables are presented as means ± standard deviations, while categorical variables are expressed as frequencies and percentages. Pearson’s chi-square test was used to detect associations between categorical variables, and Fisher’s exact test was applied in cases where expected cell frequencies were less than 5. A p-value < 0.05 was considered statistically significant. Quantitative variables, including HbA1c, BMI, and lipid markers, were analyzed both as continuous and categorical values according to clinical thresholds. Records missing essential biochemical or anthropometric indicators were excluded list-wise from final statistical testing. Selection bias was minimized by including all eligible adults screened during the study period.

Ethical considerations

This cross-sectional study was approved by the Regional Research Ethics Committee - Qassim Health Cluster (IRB: 607/46/8768). The study was conducted in accordance with the ethical principles of the Declaration of Helsinki for research involving human subjects. Given that the data were extracted from the electronic HIS without direct patient contact, the requirement for written informed consent was waived by the ethics committee. All patient records were anonymized prior to analysis to ensure confidentiality, in alignment with the Saudi National Committee of Bioethics regulations.

## Results

Demographic characteristics of participants

The study analyzed 7,359 participants with a mean age of 38 ± 12.4 years (median: 38 years), ranging from 18 to 74 years, screened across Qassim's PHCs. The first quartile (Q1) for age was 27 years, indicating that 25% of the individuals in the dataset were 27 years old or younger. The gender distribution showed a predominance of females (4,402, 59.8%) compared to males (2,957, 40.2%) within the cohort (Table 1).

**Table 1:** Demographic and Anthropometric Characteristics of Study Participants

Characteristic	(N = 7,359)
Age (years)	
Median (IQR)	38 (27-48)
Min-Max	18-74
Gender, n (%)	
Female	4402 (59.82)
Male	2957 (40.18)
BMI (kg/m^2^)	
Mean ± SD	27.03 ± 5.38

Key lipid profiles

The mean total cholesterol of 4.84 ± 0.88 mmol/L falls within the desirable range, below 5.2 mmol/L. However, 25% of the population has total cholesterol levels at or above 5.4 mmol/L, which is in the borderline high range. The mean HDL cholesterol at 1.36 ± 0.32 mmol/L is in the "better" range. However, 25% of the population has HDL levels that could be considered low. Conversely, the mean LDL cholesterol was 3.03 ± 0.80 mmol/L, exceeding the recommended ideal of below 2.6 mmol/L, and 25% of individuals have borderline high LDL levels at or above 3.5 mmol/L. The mean triglyceride levels were 1.1 ± 0.5 mmol/L, which is within the ideal range of below 1.7 mmol/L (Table 2). Further categorization of lipid status demonstrated that 2,469 (33.55%) participants had high LDL, and 2,456 (33.37%) had high total cholesterol. Low HDL was observed in 1,927 (26.19%) of the cohort, while high triglycerides were less prevalent at 1,058 (14.38%). Overall, a significant majority of the study population (4,389, 59.64%) was diagnosed with dyslipidemia (Table 3).

**Table 2:** Descriptive Statistics of Key Metabolic and Cardiovascular Markers HDL: high-density lipoprotein; LDL: low-density lipoprotein; BP: blood pressure; % NGSP: percentage standardized as per the National Glycohemoglobin Standardization Program

Variable	N	Mean ± SD	Q1	Q3	Clinical reference range
Total cholesterol (mmol/L)	7359	4.84 ± 0.88	4.22	5.41	<5.2 mmol/L
HDL cholesterol (mmol/L)	7359	1.36 ± 0.32	1.11	1.57	≥1.55 mmol/L
LDL cholesterol (mmol/L)	7359	3.03 ± 0.80	2.46	3.59	<2.6 mmol/L
Triglyceride (mmol/L)	7359	1.11 ± 0.51	0.72	1.39	<1.7 mmol/L
Diastolic BP (mmHg)	7359	72.65 ± 8.16	67	80	<80 mmHg
Systolic BP (mmHg)	7359	118.51 ± 8.09	112	123	<120 mmHg
HbA1c (% NGSP)	7359	5.66 ± 0.98	5.2	5.75	<5.7%

**Table 3:** Prevalence of Selected Health Conditions and Lifestyle Factors HDL: high-density lipoprotein; LDL: low-density lipoprotein; HbA1c: glycated hemoglobin

Health Condition/Factor	Count	Percentage (%)
High LDL	2469	33.55
High total cholesterol	2456	33.37
High triglycerides	1058	14.38
Low HDL	1927	26.19
Overall dyslipidemia	4389	59.64
Obesity (BMI > 30)	2039	27.71
Diabetes (HbA1c > 6.5%)	708	9.62
Severe diabetes (HbA1c > 8%)	298	4.05
Insufficient physical activity	3830	52.05
Sufficient physical activity	3529	47.95

BP characteristics

The BP measurements for the study population revealed a mean diastolic BP of 72.65 ± 8.16 mmHg, with an interquartile range (IQR) of 67 to 80 mmHg. The mean systolic BP was 118.51 ± 8.09 mmHg, with an IQR of 112 to 123 mmHg (Table 2). Comparing these to the normal BP definition of less than 120/80 mmHg, the average values generally fell within the healthy range. Notably, the analysis for extreme BP values showed that none of the 7,359 participants had a systolic BP greater than 150 mmHg or a diastolic BP greater than 100 mmHg.

HbA1c levels and diabetes prevalence

The HbA1c levels of the 7,359 participants showed a mean of 5.66 ± 0.98%, with the first quartile (Q1) at 5.20% and the third quartile (Q3) at 5.75%. Based on established clinical thresholds, 708 (9.62%) of the cohort had an HbA1c greater than 6.5%, indicative of a diabetes diagnosis. Furthermore, a subset of these individuals (298, 4.05%) exhibited an HbA1c greater than 8%, suggesting poorly controlled diabetes (Table 3).

BMI and physical activity levels

The study population exhibited a mean BMI of 27.03 ± 5.38 kg/m^2^ (Table 1), with an IQR spanning from 23.30 to 30.46. Classification based on BMI thresholds revealed that 2,039 (27.71%) participants were categorized as obese (BMI > 30 kg/m^2^). Regarding physical activity, a majority of participants (3,830, 52.05%) reported insufficient levels of activity, while 3,529 (47.95%) met the recommended guidelines (Table 3).

Association between physical activity and HbA1c

A chi-square test of independence was conducted to examine the association between physical activity levels and HbA1c diagnosis (HbA1c > 6.5%). The contingency table revealed that among those with insufficient physical activity, 3,461 had normal HbA1c and 369 had HbA1c > 6.5%. Among those with sufficient physical activity, 3,190 had normal HbA1c and 339 had HbA1c > 6.5%. The chi-square test yielded a Pearson chi-square value of 0.002 with 1 degree of freedom, resulting in a p-value of 0.967. This indicates that there is no statistically significant association between physical activity levels and HbA1c-defined diabetes (HbA1c > 6.5%) in the study population (Table 4).

**Table 4:** Chi-Square Test Results for Associations With Physical Activity

Variable	Insufficient Activity (n = 3830)	Sufficient Activity (n = 3529)	Chi-Square	P-value
Diabetes diagnosis (HbA1c)				
Normal (≤6.5%)	3461	3190	0.002	0.967
Diabetes (>6.5%)	369	339		
BMI status				
Normal (≤30 kg/m^2^)	2733	2587	3.484	0.062
Obese (>30 kg/m^2^)	1097	942		
Gender				
Female	2322	2080	2.173	0.140
Male	1508	1449		

Association between physical activity and BMI

A chi-square test of independence was conducted to assess the association between physical activity levels and BMI status (BMI > 30 kg/m^2^). The analysis revealed that among participants with insufficient physical activity, 2,733 had a normal BMI and 1,097 were categorized as obese. For those reporting sufficient physical activity, 2,587 had a normal BMI while 942 were obese. The Pearson chi-square value was 3.484 with 1 degree of freedom, yielding a p-value of 0.062. This result indicates that the association between physical activity level and BMI > 30 kg/m^2^ did not reach conventional statistical significance (p < 0.05) in this study population (Table 4).

Association between physical activity and gender

A chi-square test of independence was performed to assess the association between physical activity levels and gender. The contingency table revealed that 2,322 females and 1,508 males reported insufficient physical activity, while 2,080 females and 1,449 males reported sufficient physical activity. The chi-square test yielded a Pearson chi-square value of 2.173 with 1 degree of freedom, resulting in a p-value of 0.140. This finding indicates that there was no statistically significant association between physical activity level and gender in the study population (Table 4).

## Discussion

The current study, conducted within the framework of the SMoC screening program in Qassim, successfully uncovered a substantial burden of previously undiagnosed cardiometabolic risk factors among a cohort of 7,359 adults. Despite a relatively young mean age of 38 years, our findings highlight significant public health concerns, particularly regarding dyslipidemia, prediabetes/diabetes, and obesity.

Our analysis of lipid profiles revealed a critical insight: while average total cholesterol and triglyceride levels appeared within desirable ranges, a substantial proportion of the population exhibited concerning lipid abnormalities. Specifically, approximately 25% of participants had total cholesterol levels at or above 5.4 mmol/L (borderline high), and 25% had LDL-cholesterol levels at or above 3.5 mmol/L (borderline high). Crucially, nearly 60% of the study population was diagnosed with overall dyslipidemia, encompassing various lipid abnormalities such as high LDL (2,469, 33.6%) and high total cholesterol (2,456, 33.4%). This is alarmingly higher than recent national screening data showing dyslipidemia affects 25-40% of Saudi adults [13]. This high prevalence, even in a cohort with a mean age of 38, highlights a widespread suboptimal LDL cholesterol management and suggests the importance of early screening and intervention strategies, including the potential consideration of statin therapy, per Saudi lipid management guidelines [14], for a significant segment of this population to mitigate future cardiovascular risks. These findings align with broader epidemiological trends in Saudi Arabia, where dyslipidemia affects a considerable portion of the adult population.

Conversely, the BP findings presented a generally healthy cardiovascular profile, with mean systolic and diastolic BPs falling within the normal range. Notably, the absence of individuals with severe hypertension (systolic BP > 150 mmHg or diastolic BP > 100 mmHg) was a crucial observation. The complete absence of individuals presenting with severe hypertension (BP > 150/100 mmHg) is a notable finding that reflects the inherent selection criteria of the screening cohort. The sample comprised relatively young (mean age 38.0 years), asymptomatic individuals attending PHCs for routine wellness evaluations. Patients experiencing symptomatic or severe hypertensive crises systematically bypass routine preventative screening programs, presenting directly to urgent care or emergency departments. This pathway inherently creates a selection bias toward normotensive or mildly hypertensive presentations within this specific primary care dataset. This unexpected dissociation - a high prevalence of dyslipidemia coupled with low prevalence of severe hypertension - aligns with regional patterns where dyslipidemia often precedes hypertension in Gulf and Saudi populations [15], and suggests potential differences in underlying etiologies or risk factor profiles, or perhaps more effective preventive measures for BP management in this specific population. Further research could explore factors contributing to this dichotomy, such as unique lifestyle patterns, a younger cohort, or genetic predispositions.

The HbA1c findings revealed a concerning prevalence of undiagnosed diabetes, with 708 (9.6%) participants meeting the diagnostic criteria for diabetes (HbA1c > 6.5%), which is lower than earlier regional estimates of 30-58% undiagnosed cases [16,17]. Furthermore, a subset of 298 (4.1%) participants exhibited HbA1c > 8%, indicating potentially unmanaged or severe diabetes and a heightened risk for long-term complications. This significant metabolic challenge, especially when considered alongside the high prevalence of dyslipidemia, points to a potential clustering of cardiometabolic risk factors within the Qassim population. This aligns with national data indicating a surge in diabetes prevalence across Saudi Arabia [18].

Regarding anthropometric measures and lifestyle, the study population exhibited a mean BMI of 27.03 ± 5.38 kg/m^2^, falling into the overweight category. Alarmingly, 2,039 (27.7%) participants were classified as obese (BMI > 30 kg/m^2^), which is slightly lower than the local average obesity prevalence of 35%, yet notably higher than the global obesity prevalence of 13% [19]. Over half of this cohort (3,830, 52.1%) reported insufficient physical activity, mirroring the local national trends of physical activity at 57.8% [20]. This sedentary lifestyle, coupled with high rates of overweight and obesity, strongly correlates with the observed prevalence of dyslipidemia and diabetes, reinforcing the intertwined nature of these metabolic risk contributors in this population [21]. These findings highlight substantial lifestyle-related health challenges that mirror the rapid epidemiological transition and rising NCD burden seen across Gulf Cooperation Council countries due to urbanization and dietary shifts. Addressing these through targeted public health interventions focusing on diet and exercise is crucial for improving overall metabolic health and reducing the incidence of NCDs.

Intriguingly, while previous studies in Saudi populations have identified an association between physical inactivity and diabetes [22], the statistical analyses in this research did not establish a statistically significant association between broad physical activity levels and HbA1c-defined diabetes (p = 0.967). Similar trends were observed in a Japanese population of 55,469 men and women who had a weak inverse association (p = 0.049) between physical activity and HbA1c [23]. Similarly, the association between physical activity and obesity (BMI > 30 kg/m^2^) was borderline non-significant (p = 0.062). These unexpected findings warrant careful consideration. While physical activity is widely recognized for its role in glucose metabolism and weight management, the lack of statistical significance in this cross-sectional study could be attributed to several factors. The binary categorization of "sufficient" versus "insufficient" physical activity may be too simplistic to capture nuanced benefits or risks. Furthermore, unmeasured confounding variables such as detailed dietary intake, genetic predispositions, or specific types and intensities of activity could be masking underlying relationships. It is also plausible that in this specific cohort, other factors, such as dietary patterns, play a more dominant role in HbA1c status or BMI. These results highlight the complexity of metabolic health and underscore the need for more granular data on lifestyle factors and multivariate analyses in future research to elucidate intricate relationships. The findings may be generalizable to primary-care-attending adults in Qassim, although external extrapolation beyond the region should be approached with caution. Replication across other demographic strata and provincial contexts would strengthen external validity.

Study limitations

This research, while providing valuable insights into the metabolic and lifestyle health profiles of the Qassim population, is subject to several limitations inherent to its design and data collection. Foremost, its cross-sectional design precludes the establishment of causal relationships or the inference of disease progression over time; thus, we can describe associations but not determine cause and effect.

Secondly, the reliance on broad categorical definitions for lifestyle factors, particularly physical activity (sufficient/insufficient), may have limited the ability to detect more nuanced and clinically relevant associations with metabolic outcomes. This limitation is evidenced by the non-significant chi-square findings for HbA1c and the borderline result for BMI. More detailed and quantitative measures of physical activity (e.g., metabolic equivalent of task (MET) hours, specific types of exercise, duration) would provide a more comprehensive understanding.

Furthermore, the absence of data on crucial confounding variables significantly restricts the depth of multivariate analysis and the ability to control for important determinants of health, such as detailed dietary intake, smoking, other medications used for pre-existing conditions, socioeconomic status indicators, family medical history of NCDs, inflammatory markers (e.g., C-reactive protein), and stress and mental health. Additionally, the analytical scope of this cross-sectional report primarily targeted physical activity as a key modifiable lifestyle determinant. Consequently, exhaustive cross-tabulations of baseline demographic predictors (e.g., age and gender predictive modeling against specific biomarkers) were not performed. Future multivariate studies and longitudinal study designs are required to fully elucidate these specific demographic risk associations and provide a more robust understanding of chronic disease epidemiology in the region.

## Conclusions

The population-level screening initiative in the Qassim region successfully uncovered a substantial hidden burden of cardiometabolic risk factors, particularly dyslipidemia, undiagnosed diabetes, and obesity, among apparently healthy adults. These findings highlight a critical gap in early disease identification within primary healthcare settings and underscore the limitations of relying solely on symptom-triggered testing, even in relatively young demographic groups.

Transitioning toward proactive, routine screening is essential to mitigate the escalating long-term cardiovascular and metabolic disease burden. Ultimately, this approach provides a strategic foundation for targeted public health interventions, lifestyle modification programs, and efficient resource allocation. Future research incorporating granular lifestyle data and longitudinal designs is warranted to fully elucidate the pathways contributing to these metabolic disparities and to inform precise preventive strategies.
